# Supplementary material for: A mapping framework of competition–cooperation QTLs that drive community dynamics
Source: Nat Commun. 2018 Aug 1;9:3010. doi: 10.1038/s41467-018-05416-w (PMC6070507; doi:10.1038/s41467-018-05416-w)
Supplement: Supplementary file 1 — Supplementary Information [file 41467_2018_5416_MOESM1_ESM.pdf]

A mapping framework of competition-cooperation QTLs that drive community dynamics

Jiang et al.

Supplementary Tables

Supplementary Table 1 The estimates of growth parameters (and their standard errors) for microbial abundance by Gompertz (G), logistic (L), and Richards (R) equations in monoculture and by these three equations and Lotka-Volterra (LV) equations in co-culture. According to F-test, the optimal equation is the R for both bacterial species in monoculture, since F-values for both G and L are significant (see the text). The AIC values calculated identify the LV as optimal equations for both species in co-culture.

Monoculture

	E. coli			*S. aureus*		
Parameter	G	L	R	G	L	R
A	16.07±2.12	16.41±1.23	17.41±1.59	18.12±2.82	17.85±2.32	18.61±1.67
r	0.99±0.11	1.07±0.17	1.95±0.27	0.76±0.13	0.65±0.12	1.52±0.31
λ	16.07±2.12	-2.33±0.21	-0.71±0.06	-5.43±0.65	-7.01±1.02	-2.02±0.26
v	-	-	-0.99±0.14	-	-	-0.93±0.08
F-value	10.35**	18.21**	-	4.60*	7.78*	-

Co-culture

Parameter	G		L		R		LV	
	E. coli	*S. aureus*	*E. coli*	*S. aureus*	*E. coli*	*S. aureus*	*E. coli*	*S. aureus*
A	15.67±2.35	13.03±2.0	15.51±1.71	12.91±1.68	16.07±1.83	13.22±1.29	-	-
r	1.23±0.25	1.01±0.13	1.12±0.15	0.92±0.16	1.88±0.30	1.61±0.17	-	-
λ	-1.71±0.15	-1.75±0.3	-2.01±0.24	-2.07±0.37	-0.65±0.08	-0.58±0.05	-	-
v	-	-	-	-	-0.93±0.11	-0.92±0.06	-	-
r_e (r_s)	-	-	-	-	-	-	0.28±0.06	0.25±0.03
$\alpha_{e\|s}$ ($\alpha_{s\|e}$)	-	-	-	-	-	-	2.77±0.61	1.73±0.32
K_e (K_s)	-	-	-	-	-	-	83.1±8.12	62.35±8.27
AIC	18.22		25.78		17.21		**15.92**	

*Significant at the 5% level, **Significant at the 1% level.

Supplementary Table 2 Gene annotations of significant QTLs detected from different species in co-culture and monoculture by competition-cooperation mapping (CoCoM) and functional mapping (FunMap). There are a total of 54 significant pairs (in boldface) between 41 QTLs from *E. coli* and 12 QTLs from *S. aureus*.

**Co-culture (CoCoM)**							**Co-culture (CoCoM)**						
E. coli				***S. aureus***			***E. coli***				***S. aureus***		
**Pair**	**QTL**	**Position**	**Gene**	**Gene**	**Position**	**QTL**	**Pair**	**QTL**	**Position**	**Gene**	**Gene**	**Position**	**QTL**
**1**	1	E19056	*nhaR*	*ggt*	S188004	1	**38**	30	E3056867	-	*spIA*	S867513	11
**2**	2	E49080	*kefC*	*ggt*	S188004	1	**39**	31	E3218576	*aer*	*hp*	S342015	3
**3**	3	E62059	*rapA*	*ggt*	S188004	1	**40**	31	E3218576	*aer*		S1612255	2
**4**	4	E126510	*aceF*	*ggt*	S188004	1	**41**	32	E3228896	*ygjK*	*ggt*	S188004	1
**5**	5	E470127	*mdlA*	*ggt*	S188004	1	**42**	33	E3328548	*greA*	*hp*	S342015	3
**6**	6	E633990	*ybdL*	*ggt*	S188004	1	**43**	34	E3539826	-	-	S1612255	2
**7**	7	E759230	*sucA*	-	S1612255	2	**44**	35	E3539840	-	-	S323313	12
**8**	8	E832164	*rhlE*	*ggt*	S188004	1	**45**	36	E3544071	-	*hp*	S56698	8
**9**	9	E866870	*iaaA*	*ggt*	S188004	1	**46**	36	E3544071	-	*hp*	S2076600	10
**10**	10	E1098633	*ghrA*	*ggt*	S188004	1	**47**	37	E3956003	*ilvA*	*ggt*	S188004	1
**11**	10	E1098633	*ghrA*	*hp*	S342015	3	**48**	38	E4093625	*rhaD*	*ggt*	S188004	1
**12**	11	E1317124	*trpA*	*ggt*	S188004	1	**49**	39	E4127415	*yiiX*	*hp*	S56698	8
**13**	12	E1367778	*pspF*	*ggt*	S188004	1	**50**	39	E4127415	*yiiX*	*ebp*	S1454572	7
**14**	12	E1367778	*pspF*	-	S337827	4	**51**	39	E4127415	*yiiX*	*hp*	S2076600	10
**15**	13	E1483999	*hrpA*	*ggt*	S188004	1	**52**	40	E4320079	*phnI*	*ggt*	S188004	1
**16**	14	E1486303	*hrpA*	*ggt*	S188004	1	**53**	40	E4320079	*phnI*	-	S337827	4
**17**	15	E1827957	*astB*	*hp*	S342015	3	**54**	41	E4614704	*yjjW*	*ggt*	S188004	1
**18**	16	E1838489	*ynjC*	-	S1612255	2							
**19**	17	E1920546	*rsmF*	*hp*	S342015	3	**Monoculture (FunMap)**						
**20**	18	E1997109	*yecC*	-	S119378	4		1	E35166	*caiE*	*hp*	S341983	1
**21**	18	E1997109	*yecC*	*hp*	S289329	5		2	E171740	-	*tIrms*	S399836	2
**22**	18	E1997109	*yecC*	-	S936893	6		3	E248433	-	*hp*	S1397941	3
**23**	18	E1997109	*yecC*	*ebp*	S1454572	7		4	E669409	*cobC*	-	S1873926	4
**24**	19	E2002296	*fliC*	*ggt*	S188004	1		5	E1305980	*oppF*	*hp*	S1890216	5
**25**	20	E2206895	*yehM*	-	S936893	6		6	E1371620	*ycjM*			
**26**	21	E2233954	-	*ggt*	S188004	1		7	E1376541	*ycjR*			
**27**	22	E2252539	*yeiI*	*ggt*	S188004	1		8	E1985289	*araF*			
**28**	23	E2252542	*yeiI*	*ggt*	S188004	1		9	E2309274	*alkB*			
**29**	24	E2252925	*nupX*	*ggt*	S188004	1		10	E2842669	*hycI*			
**30**	25	E2568153	-	*ggt*	S188004	1		11	E2902468	*ygcE*			
**31**	26	E2594191	*tmcA*	*hp*	S56698	8		12	E3237931	*ygjR*			
**32**	26	E2594191	*tmcA*	*ebp*	S1454572	7		13	E3255253	-			
**33**	26	E2594191	*tmcA*	*hp*	S2076600	9		14	E3391828	*tldD*			
**34**	27	E2789300	*csiD*	-	S936893	6		15	E4056618	-			
**35**	28	E2830623	*gutQ*	*hp*	S2076600	10		16	E4569338	-			
**36**	29	E3044738	*bglA*	*ggt*	S188004	1		17	E4605923	*yjjZ*			
**37**	30	E3056867	-	*ggt*	S188004	1		18	E4606635	-			

The functional explanation of genes is given below:

Gene	Detailed information	Gene	Detailed information
nhaR	nhaR transcriptional activator of nhaA	*trpA*	trpA tryptophan synthase
kefC	kefC K+ efflux antiporter, glutathione-regulated	*pspF*	pspF psp operon transcriptional activator
rapA	rapA probable ATP-dependent RNA helicase	*ggt*	gamma-glutamyltranspeptidase
aceF	aceF pyruvate dehydrogenase	*hp*	hypothetical protein
mdlA	mdlA	*astB*	astB
ybdL	ybdL PLP-dependent methionine aminotransferase	*ynjC*	ynjC putative transport system permease protein
sucA	sucA 2-oxoglutarate dehydrogenase	*rsmF*	rsmF
rhlE	rhlE putative ATP-dependent RNA helicase	*yecC*	yecC putative ATP-binding component of a transport system
iaaA	iaaA putative asparaginase	*ebp*	elastin binding protein
ghrA	ghrA putative dehydrogenase	*nupX*	nupX putative pyrimidine nucleoside transporter
hrpA	hrpA helicase, ATP-dependent	*tmcA*	tmcA
fliC	fliC flagellar biosynthesis	*spIA*	signal peptidase IA
yehM	yehM	*aer*	aer aerotaxis sensor receptor
yeiI	yeiI putative kinase	*ygjK*	ygjK putative isomerase
csiD	csiD	*greA*	greA transcription elongation factor
gutQ	gutQ	*ilvA*	ilvA threonine deaminase
bglA	bglA 6-phospho-beta-glucosidase A	*rhaD*	rhaD rhamnulose-phosphate aldolase
yiiX	yiiX	*tIrms*	type I restriction-modification system subunit M
phnI	phnI phosphonate metabolism	*cobC*	cobC homolog of Salmonella cobC
yjjW	yjjW putative activating enzyme	*oppF*	oppF homolog of Salmonella ATP-binding protein of oligopeptide ABC transport system
caiE	caiE possible synthesis of cofactor for carnitine racemase and dehydratase	*ycjM*	ycjM putative polysaccharide hydrolase
ycjR	ycjR	*ygcE*	ygcE putative kinase
araF	araF L-arabinose-binding periplasmic protein	*ygjR*	ygjR
alkB	alkB DNA repair system specific for alkylated DNA	*tldD*	tldD suppresses inhibitory activity of CsrA
hycI	hycI protease involved in processing C-terminal end of the large subunit of hydrogenase 3	*yjjZ*	yjjZ

Supplementary Table 3 Power and false positive rates (FPR) of QTL detection by functional mapping (FunMap) and competition-cooperation mapping (CoCoM) from the data simulated from these two models, respectively, under different heritabilities, 0, 0.05 and 0.10, for sample size 200 and 45.

n = 200

		Data					
		FunMap			CoCoM		
		FPR	Power		FPR	Power	
Heritability		0	0.05	0.1	0	0.05	0.1
Method	FunMap	0.09	0.82	0.95	0.08	0.15	0.23
	CoCoM	-	-	-	0.10	0.72	0.87

n = 45

		Data					
		FunMap			CoCoM		
		FPR	Power		FPR	Power	
Heritability		0	0.05	0.1	0	0.05	0.1
Method	FunMap	0.06	0.66	0.78	0.05	0.06	0.17
	CoCoM	-	-	-	0.08	0.42	0.63

Power was empirically calculated as the proportion of the number of simulation replicates in which significant QTLs were detected over the total number of simulation replicates (1000) for the data simulated under the assumption of QTL occurrence. False positive rates (FPR) were calculated as the proportion of the number of simulation replicates in which significant QTLs were detected over the total number of simulation replicates (1000) for the data simulated under the assumption of no QTL.

Supplementary Table 4 Average sequencing depth and coverage for two species.

E. coli

Sample	Strain ID	Mapping rate (%)	Average sequencing depth	Coverage (%) ≥ 20X (%）
D1	1.2527(CGMCC)	84.22	125	93.58
D2	1.1583(CGMCC)	82.76	182	99.98
D3	1.1570(CGMCC)	87.66	131	99.86
D4	1.487(CGMCC)	86.14	172	90.94
D5	1.1571(CGMCC)	87.00	230	99.02
D6	1.1595(CGMCC)	94.04	101	97.50
D7	1.1280(CGMCC)	83.56	87	93.48
D8	1.2463(CGMCC)	85.58	127	92.97
D9	1.2389(CGMCC)	71.75	123	85.84
D10	1.2385(CGMCC)	66.87	85	85.20
D11	1.1739(CGMCC)	99.23	163	98.30
D12	1.1645(CGMCC)	89.59	190	99.85
D13	1.365(CGMCC)	89.58	224	99.22
D14	1.2154(CGMCC)	74.41	117	91.96
D15	1.1747(CGMCC)	73.32	103	95.74
D16	1.1740(CGMCC)	93.83	124	98.30
D17	1.1632(CGMCC)	68.19	112	84.64
D18	1.1636(CGMCC)	92.20	191	99.85
D19	1.1566(CGMCC)	88.62	131	97.44
D20	1.1557(CGMCC)	91.48	115	93.56
D21	1.1499(CGMCC)	84.76	187	93.66
D22	1.1126(CGMCC)	82.38	179	99.99
D23	1.1102(CGMCC)	84.45	173	93.59
D24	1.1746(CGMCC)	68.86	94	84.55
D25	10047(CICC)	66.50	77	82.95
D26	10221(CICC)	72.60	118	99.84
D27	10222(CICC)	95.14	220	99.28
D28	10223(CICC)	98.50	194	99.73
D29	10224(CICC)	96.69	166	98.31
D30	10242(CICC)	98.55	177	98.66
D31	10245(CICC)	94.75	133	98.89
D32	10247(CICC)	95.08	133	98.29
D33	1.2810(CGMCC)	92.30	201	99.33
D34	1.129(CGMCC)	81.90	119	91.83
D35	10250(CICC)	94.98	124	99.11
D36	1.1574(CGMCC)	84.72	74	98.99
D37	1.505(CGMCC)	67.50	95	82.75
D38	1.747(CGMCC)	94.28	256	99.67

D39	1.881(CGMCC)	90.25	241	93.67
D40	10003(CICC)	82.87	156	92.07
D41	1.3373(CGMCC)	82.23	121	93.83
D42	1.8723(CGMCC)	69.66	110	84.56
D43	10032(CICC)	74.35	131	89.55
D44	10243(CICC)	99.11	148	98.17
D45	1.2170(CGMCC)	91.04	148	99.83
Mean		85.19±9.90	146.84±45.79	94.85±5.44

S. aureus

**Sample**	**Strain ID**	**Mapping rate (%)**	**Average sequencing depth**	**Coverage(%)≥ 20X（%）**
J1	21676(CICC)	74.88	218	96.47
J2	21600(CICC)	22.88	76	94.49
J3	01334(ACCC)	87.28	281	94.79
J4	01340(ACCC)	87.56	348	90.58
J5	01332(ACCC)	86.40	333	94.78
J6	01331(ACCC)	85.43	320	90.51
J7	01339(ACCC)	86.49	303	90.48
J8	10341(CFCC)	90.13	367	94.98
J9	1.8721(CGMCC)	91.20	300	94.25
J10	1.1697(CGMCC)	87.39	332	92.59
J11	1.1476(CGMCC)	67.42	396	93.98
J12	141396(CPCC)	91.11	362	93.55
J13	140594(CPCC)	93.20	287	93.46
J14	140575(CPCC)	91.27	360	93.56
J15	21648(CICC)	91.50	299	92.97
J16	10786(CICC)	89.12	289	93.62
J17	22942(CICC)	86.20	310	92.96
J18	AB94004(CCTCC)	90.68	341	93.96
J19	AB91093(CCTCC)	93.57	318	93.52
J20	AB91053(CCTCC)	93.92	365	93.45
J21	23699(CICC)	17.93	54	92.76
J22	23656(CICC)	86.17	312	90.21
J23	22944(CICC)	65.18	197	92.91
J24	J24*	91.95	426	93.47
J25	1.2386(CGMCC)	85.93	471	90.24
J26	10201(CICC)	34.06	96	93.13
J27	J27*	92.38	376	93.45
J28	J28*	86.60	442	92.92
J29	141405(CPCC)	92.35	359	93.6
J30	26003 (CMCC)	61.05	223	93.44

J31	26112 (CMCC)	83.56	452	90.73
J32	J32*	85.33	401	90.16
J33	26001(CMCC)	90.82	316	93.53
J34	141431(CPCC)	99.10	333	99.95
J35	140660(CPCC)	95.03	459	99.95
J36	J36*	88.21	253	90.71
J37	ATCC43300	80.32	310	87.86
J38	J38*	84.03	373	88.69
J39	J39*	90.07	385	91.14
J40	P1(CAU)	89.85	324	92.73
J41	AB18(CAU)	89.54	336	91.44
J42	CD1(CAU)	90.73	362	92.53
J43	CD9(CAU)	88.74	283	92.48
J44	CD7(CAU)	90.84	388	92.55
J45	J45*	92.96	337	94.97
Mean		83.12±17.45	321.62±89.45	92.99±2.31

Note: CGMCC: China General Microbiological Culture Collection Center. CICC: China Center of Industrial Culture Collection. ACCC: Agricultural Culture Collection of China. CFCC: China Forestry Culture Collection Center. CPCC: China Pharmaceutical Culture Collection. CCTCC: China Center for Type Culture Collection CMCC: National Center for Medical Culture Collections. CAU: China Agricultural University. Strains with "*" were isolated by our lab.

Supplementary Figures

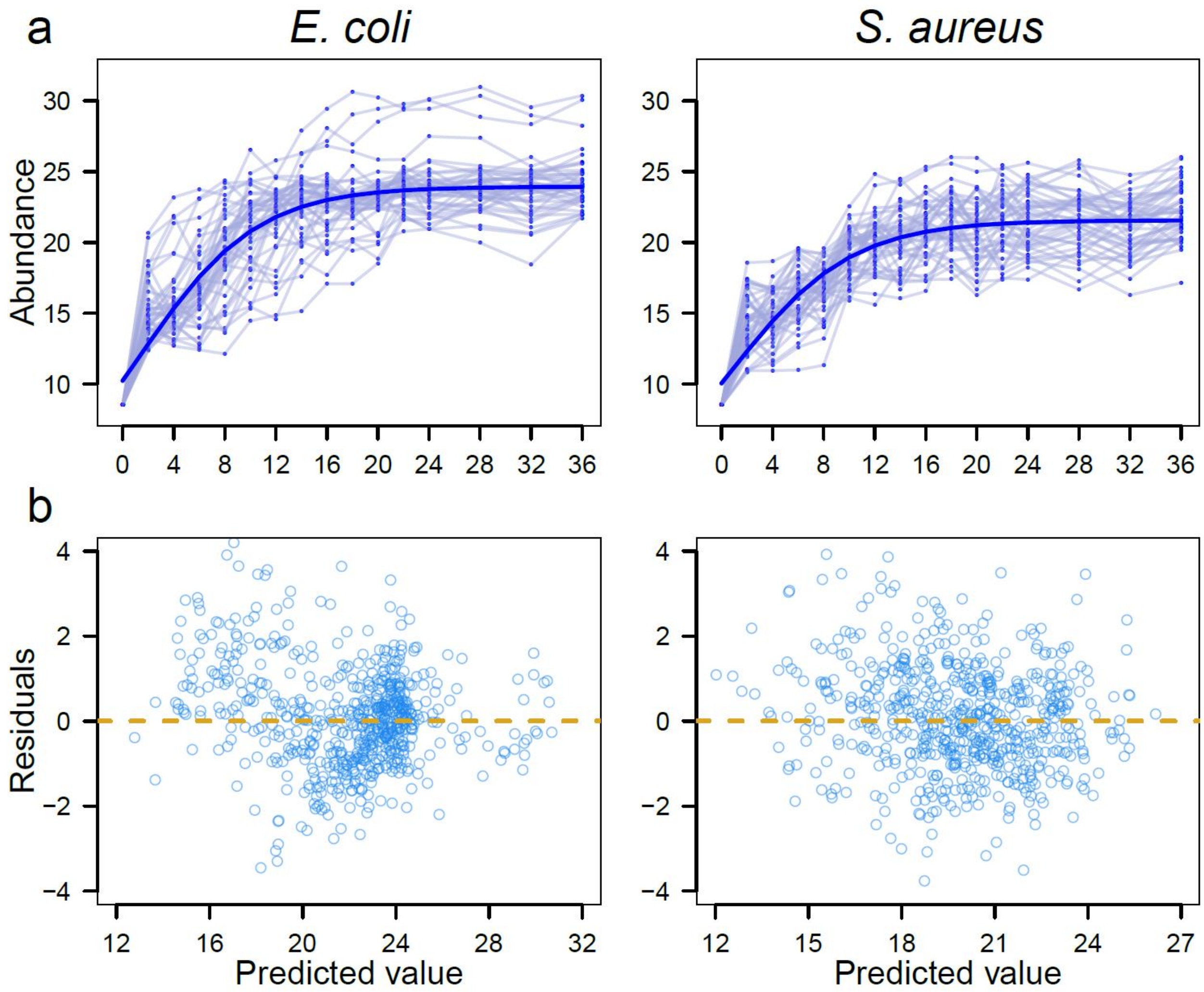

Supplementary Figure 1 Goodness-of-fit of the LV equation (2) to observational abundance data for *E. coli* and *S. aureus* in co-culture. a, the fitted mean curve (thick line) of all strains for each species, with raw data shown by grey lines. **b**, random scatters of residuals over predicted values across each strain (circle) by the ODEs, warranting the statistical behavior of data fitting.

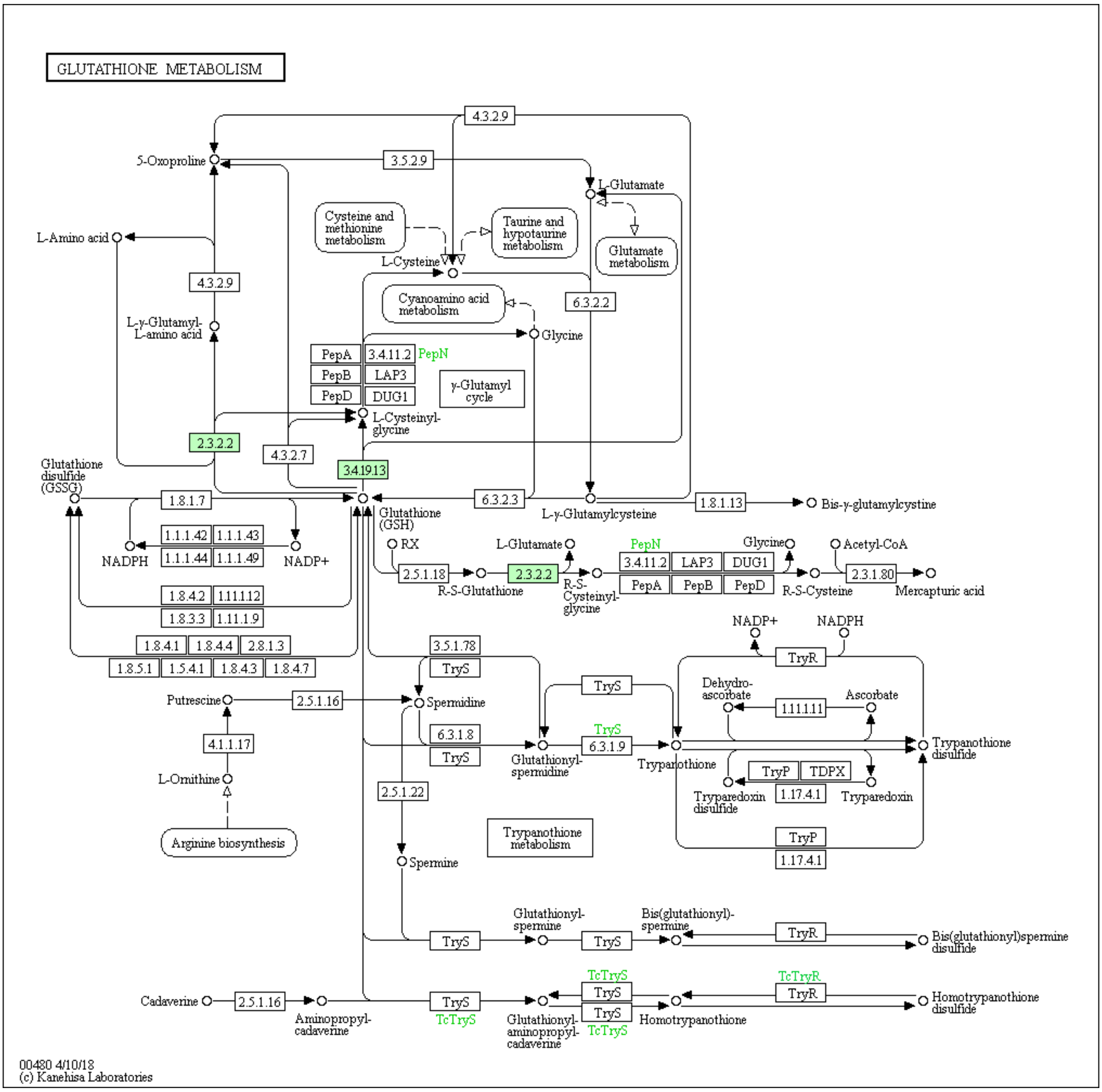

Supplementary Figure 2 The KEGG pathway map of gene *ggt*, nearby aggressive QTL S188004 detected in *S. aureus* by CoCoM. *ggt* is the gene encoding gamma-glutamyltranspeptidase (EC 2.3.2.2).

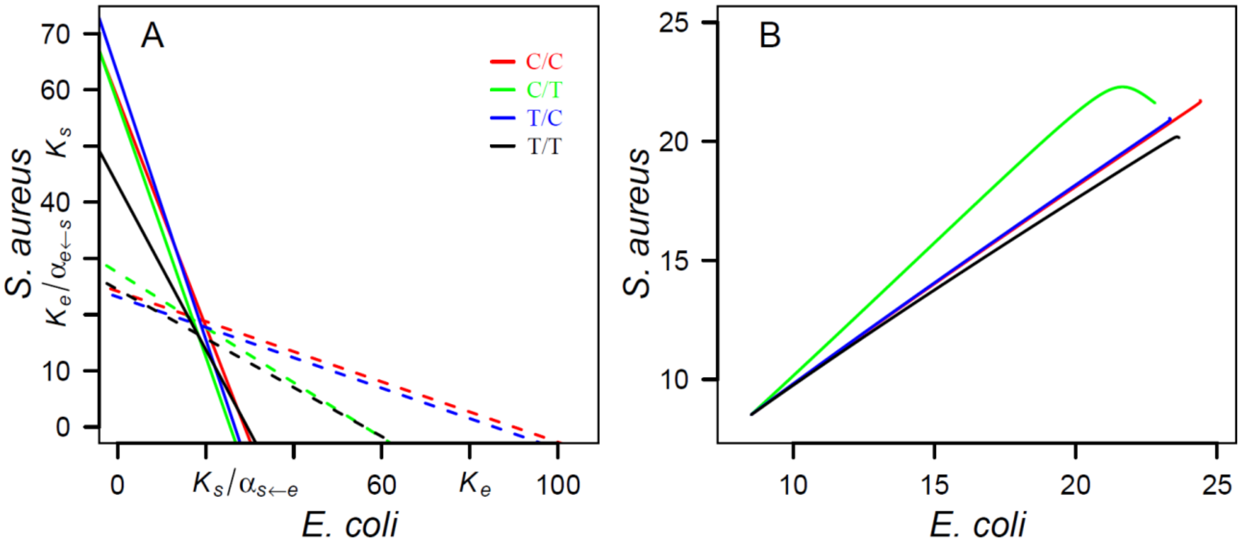

Supplementary Figure 3 States of two interacting species at four genotypic combinations C/C, C/T, T/C and T/T for E4614704 and S188004. By setting $dN_e/dt = 0$ and $dN_s/dt = 0$ to obtain zero isoclines for each species, ODE (2) charts a state-space of the combination of abundance between two species. The space has four types: (i) a predation/parasitism of *E. coli* over *S. aureus*, (ii) a predation/parasitism of *S. aureus* over *E. coli*, (iii) the cooperation between two species by benefiting from each other, and (iv) the competition by excluding from each other to exist in unstable equilibrium. **A**, plot of the abundance of *S. aureus* (solid line) against *E. coli* (slash line) at each genotypic combination. Four interspecific genotypes at QTLs E4614704 and 188004 present consistently a similar pattern of competition, but the degree of such competition varies strikingly among the genotype combinations, suggesting that these two QTLs, through their across-genome combination, are involved in the regulation of competition between *E. coli* and *S. aureus*. **B**, limit cycles of four genotypic combinations between two species.

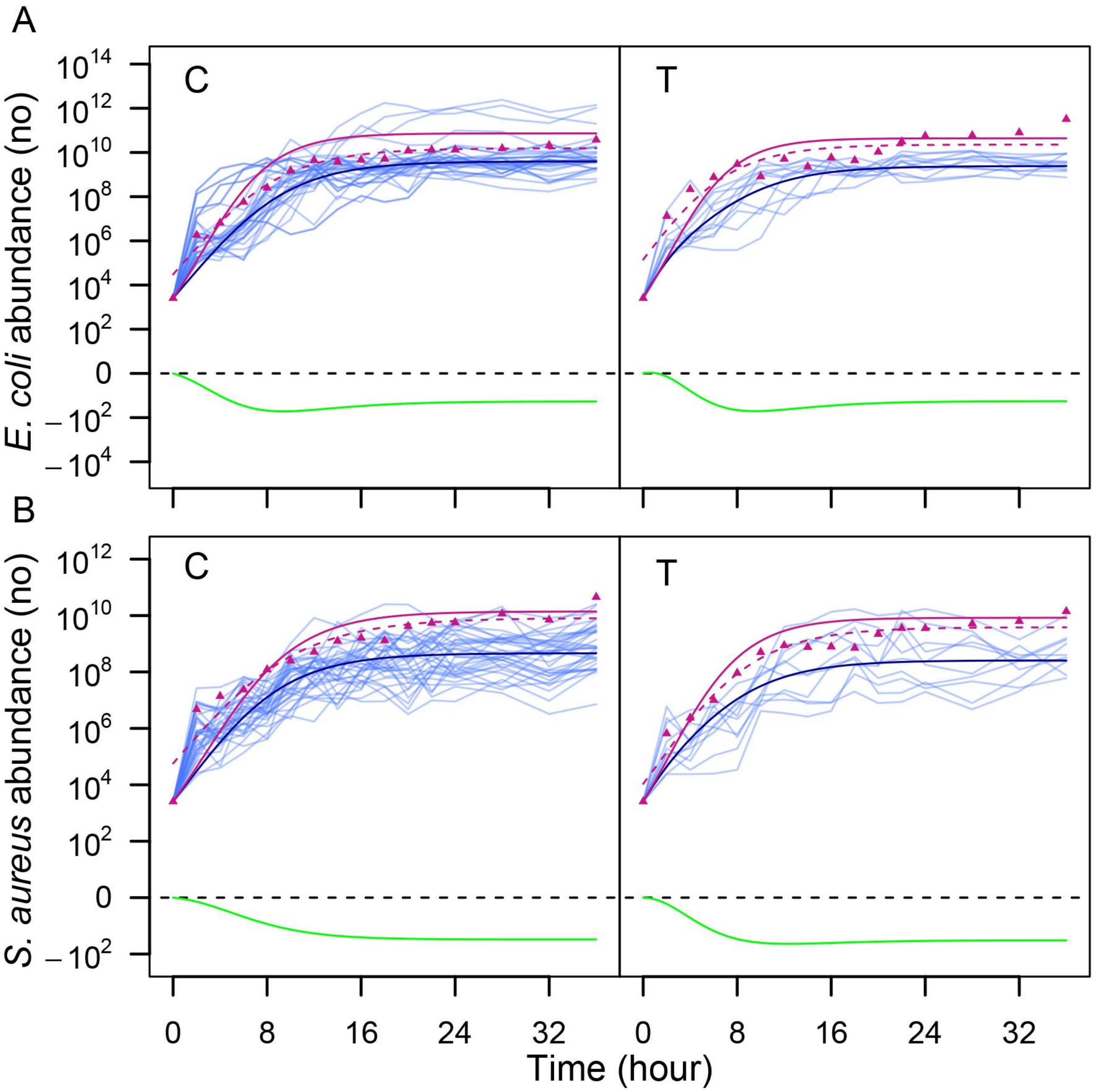

Supplementary Figure 4 Biological validation of competition-cooperation mapping (CoCoM). The overall growth of *E. coli* (**A**) and *S. aureus* (**B**) in co-culture (thick blue line) fitted to raw abundance data (thin blue lines) was decomposed into the independent growth (red solid line) and dependent growth (green line) at two alternative genotypes C and T for E4614704 and C and T for S188004. The broad agreement of the independent growth in co-culture estimated by CCM with the growth in isolation for each genotype (red triangles fitted by slash curves) suggests that the model can accurately capture the biologically grounded rules of ecological interactions.

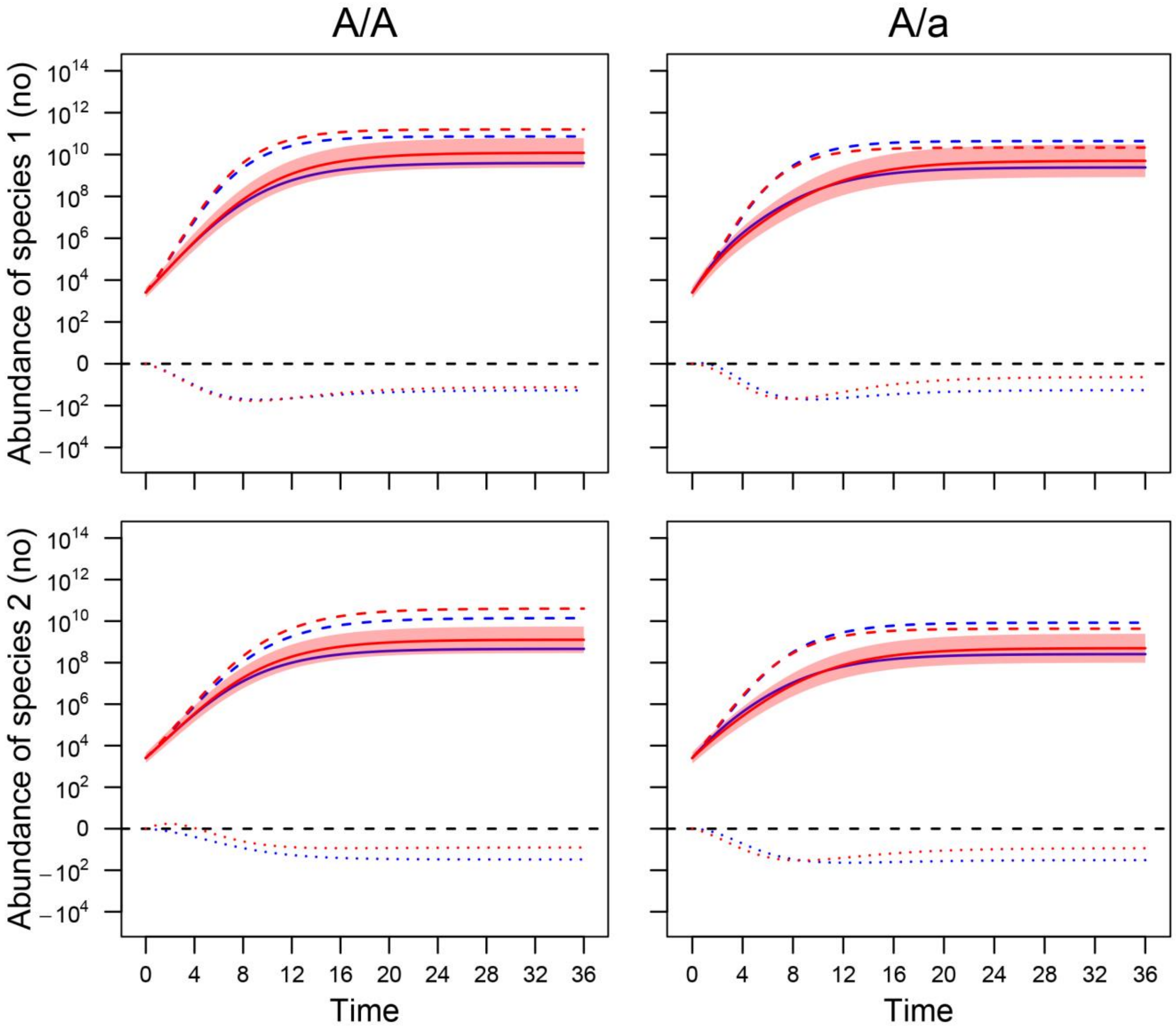

Supplementary Figure 5 Computer simulation validating the statistical properties of competition-cooperation mapping (CoCoM), as shown by a broad agreement of estimated growth curves (blue) with true growth curves (red). The overall curve, independent curve and dependent curve of each species are denoted by solid, slash and dot lines, respectively. The pink background represents the 5% confidence interval of the estimated overall curve under the sample size of 200 and heritability of 0.1.

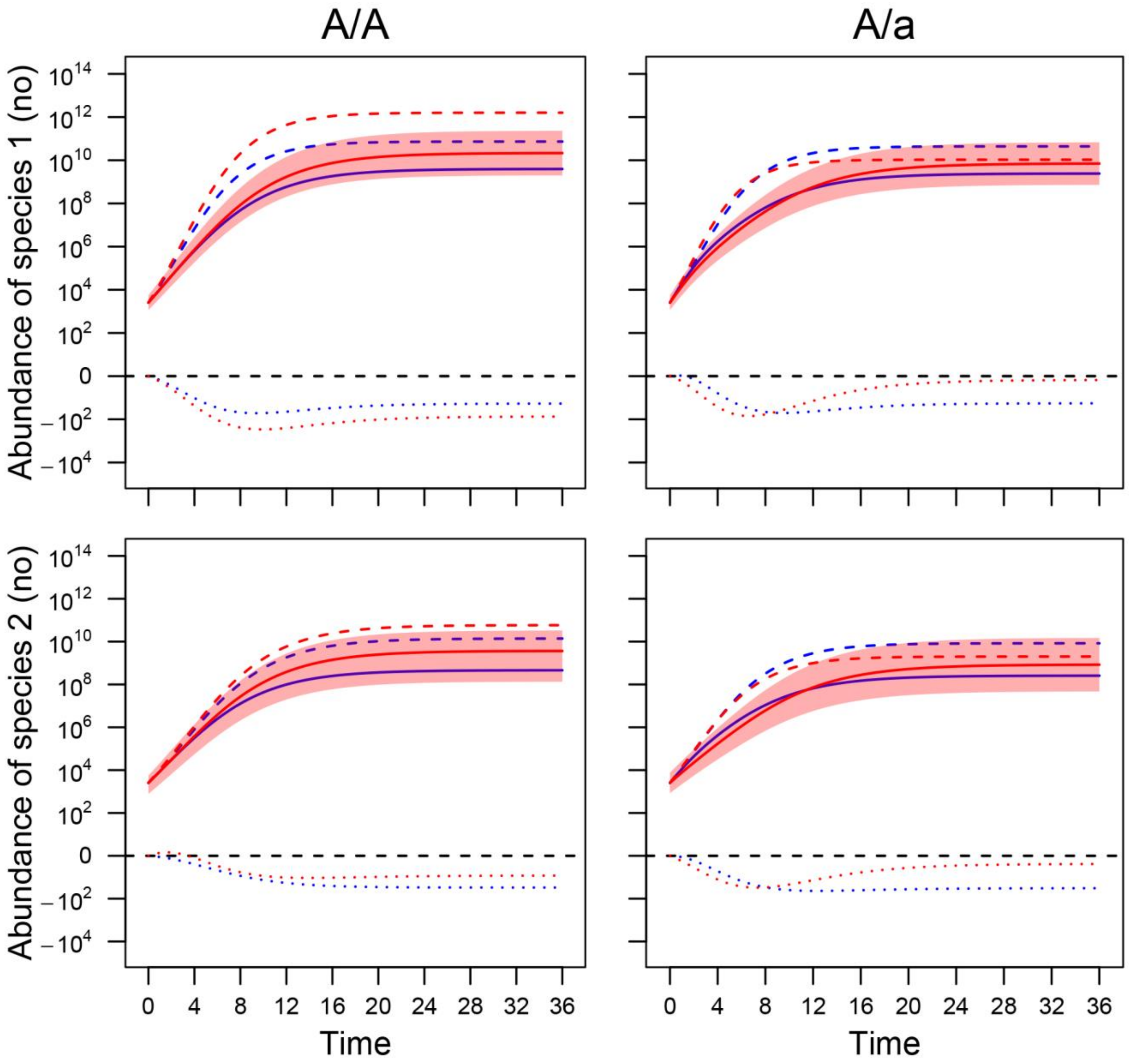

Supplementary Figure 6 Simulation results by mimicking one significant QTL pairs detected from the real example in terms of sample size, heritability and ODE parameters. The overall curve, independent curve and dependent curve of each species are denoted by solid, slash and dot lines, respectively. The pink background represents the 5% confidence interval of the estimated overall curve under the sample size of 45 and heritability of 0.1.

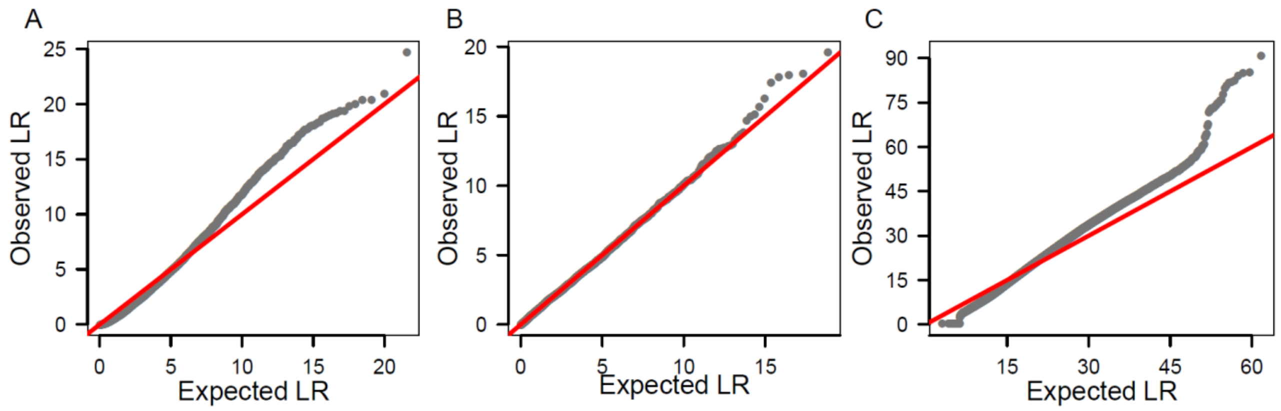

Supplementary Figure 7 Q-Q plot showing the expected distribution of association test statistics, log-likelihood ratio (LR) (x-axis), across all SNPs compared to the observed values (y-axis) by functional mapping of microbial growth for *E. coli* (**A**) and *S. aureus* (**B**) in monoculture and systems mapping of unified growth of the two species in co-culture (**C**).
